# Interferon-λ3/4 genetic variants and interferon-λ3 serum levels are biomarkers of lupus nephritis and disease activity in Taiwanese

**DOI:** 10.1186/s13075-018-1683-z

**Published:** 2018-08-29

**Authors:** Ji-Yih Chen, Chin-Man Wang, Tai-Di Chen, Yeong-Jian Jan Wu, Jing-Chi Lin, Ling Ying Lu, Jianming Wu

**Affiliations:** 1 Department of Medicine, Division of Allergy, Immunology and Rheumatology, Chang Gung Memorial Hospital, Chang Gung University College of Medicine, No. 5, Fu-Shin St. Kwei-Shan, Tao-Yuan, Taiwan; 2 Department of Rehabilitation, Chang Gung Memorial Hospital, Chang Gung University College of Medicine, No. 5, Fu-Shin St. Kwei-Shan, Tao-Yuan, Taiwan; 3 Department of Anatomic Pathology, Chang Gung Memorial Hospital, Chang Gung University College of Medicine, Tao-Yuan, Taiwan; 4 Department of Medicine, Division of Allergy Immunology and Rheumatology, Kaohsiung Veterans General Hospital, No. 386, Dazhong 1st Rd, Zuoying District, Kaohsiung City, 81362 Taiwan; 5 Department of Veterinary and Biomedical Sciences, Department of Medicine, University of Minnesota, 235B Animal Science/Vet. Med. Bldg, 1988 Fitch Avenue, St. Paul, MN 55108 USA

**Keywords:** Interferon λ, Lupus nephritis, Systemic lupus erythematosus

## Abstract

**Background:**

Type III interferons (IFNs) or IFN-λs are the newly discovered cytokines that primarily target the cells of epithelial and myeloid lineages, which are major components of kidneys. The current study aimed to investigate whether IFN-λs are involved in the pathogenesis of systemic lupus erythematosus (SLE) and lupus nephritis.

**Methods:**

TaqMan allele discrimination assays were used to determine *IFNL3/4* SNP genotypes of 1620 healthy controls and 1013 SLE patients (two independent cohorts consisting of 831 and 182 subjects, respectively) from Taiwan. The distributions of *IFNL3/4* SNP genotypes and allele frequencies were compared between SLE patients and healthy controls and among SLE patients stratified by clinical phenotypes. ELISA was used to determine the serum IFN-λ3 concentrations of SLE patients.

**Results:**

All major *IFN3/4* SNP alleles were significantly associated with the risk for lupus nephritis (rs8099917T, *P*_FDR_ = 0.0021, OR 1.75, 95% CI 1.24–2.47; rs12979860C, *P*_FDR_ = 0.0034, OR 1.65, 95% CI 1.18–2.30; rs4803217C, *P*_FDR_ = 0.0021, OR 1.76, 95% CI 1.25–2.48; and ss469415590TT, *P*_FDR_ = 0.0021, OR 1.73, 95% CI 1.23–2.42) among SLE patients. Similarly, the major *IFNL3/4* SNP haplotype rs8099917T-ss469415590TT-rs12979860C-rs4803217C (or T-TT-C-C) was a significant risk factor for lupus nephritis (*P* = 0.0015, OR 1.68, 95% CI 1.22–2.32). Additionally, all minor *IFN3/4* SNP alleles were significantly associated with SLE susceptibility in nephritis-negative SLE patients as compared to normal healthy controls (rs8099917G, *P*_FDR_ = 0.00177, OR 1.68, 95% CI 1.24–2.28; rs12979860T, P_FDR_ = 0.00299, OR 1.58, 95% CI 1.18–2.32; rs4803217A, *P*_FDR_ = 0.00176, OR 1.65, 95% CI 1.22–2.23; and ss469415590ΔG, *P*_FDR_ = 0.00176, OR 1.70, 95% CI 1.26–2.29). Furthermore, the elevated serum levels of IFN-λ3 were significantly correlated with the complement depression and the high SLE disease activities in SLE patients.

**Conclusions:**

*IFN-λ3/4* genetic variants play a unique role in the development of lupus nephritis and SLE.

**Electronic supplementary material:**

The online version of this article (10.1186/s13075-018-1683-z) contains supplementary material, which is available to authorized users.

## Background

Systemic lupus erythematosus (SLE) is a prototypic autoimmune disease resulting from abnormal immune responses of immune cells including dendritic cells (DCs), macrophages, monocytes, neutrophils, and lymphocytes [1, 2]. In addition, nonimmune cells such as endothelial, epithelial, and renal tubular cells contribute to the development of SLE [3]. Genetic studies have identified multiple genes involved in the pathogenesis of SLE. However, the functional roles of various risk genes in the development of SLE remain incompletely understood.

Type III interferons (IFNs) or IFN-λs (IFNLs) are newly discovered cytokines that mediate diverse immune functions [4]. Located at chromosome 19q13, the IFN-λ gene family consists of four newly identified members: IL-29 (IFN-λ1 or IFNL1), IL-28A (IFN-λ2 or IFNL2), IL-28B (IFN-λ3 or IFNL3), and IFN-λ4 (IFNL4). IFN-λs are mainly produced by monocytes, macrophages, DCs, and bronchial epithelial cells in response to viral infections [4]. IFN-λs bind to a distinct receptor complex (IL-28RA/IL-10Rβ) that is primarily expressed by cells of epithelial origin (respiratory, intestinal, and reproductive tract epithelial cells, hepatocytes, and keratinocytes) and myeloid linage [4]. IFN-λs exert highly circumscribed antiviral effects through intracellular activation of antiviral host factors in the infected cells, similar to the type I IFNs [5]. Accumulating evidence suggests that IFN-λs have a unique role in regulating innate and adaptive immune responses targeting microbial infections of epithelial cells expressing cognate receptor complexes [4, 6].

Type I IFNs (IFN-α, IFN-β, IFN-ε, and IFN-ω) initiate signal transduction cascades leading to expression of IFN-stimulated genes (ISGs) that control virus replication [7]. The expression of type I IFNs and type I IFN-inducible genes is significantly increased in patients with SLE, pointing to a role of type I IFNs in SLE pathogenesis [7–10]. High levels of circulating type I IFNs and type I IFN-induced cell activation are heritable traits in families with SLE, suggesting that the alleles responsible for a strong type I IFN activation pathway are risk factors for the development of SLE [11–13]. While IFN-λs mediate antiviral functions similar to the type I IFNs [4], the role of IFN-λs in the development of SLE remains unknown as the IFNL locus was not revealed by genome-wide association studies (GWAS) [14, 15]. In particular, the IFN-λ3 SNPs (rs8099917, rs12979860, and rs4803217) in strong linkage disequilibrium with the IFN-λ4 SNP rs368234815 (TT/ΔG) have been suggested to influence IFN-λ3 mRNA stability, IFN-λ3/4 expression, ISG levels, and the response to IFN-α treatment [16]. The present study was aimed to investigate whether the IFN-λ3/4 genes are associated with SLE susceptibility and disease phenotypes in Taiwanese.

## Methods

### Study participants and disease activity assessment

SLE patients were recruited at the Rheumatology Clinics of Chang Gung Memorial Hospital. All SLE patients fulfilled the 1982 and 1997 American College of Rheumatology (ACR) criteria for the classification of SLE [17]. Lupus activity was assessed according to the SLE Disease Activity Index (SLEDAI) [18], which defines SLEDAI > 4 as high SLE disease activity. Ethnically matched healthy controls were recruited following a questionnaire survey to ensure that the control subjects were free of any autoimmune diseases. The human study was approved by the ethics committees of Chang Gung Memorial Hospital. All subjects provided written consent to participate in human studies according to the Declaration of Helsinki.

### Genomic DNA extraction

Genomic DNA was extracted from anticoagulated peripheral blood using the Gentra Puregene DNA isolation kit.

### SNP genotype assays

Validated made-for-order TaqMan SNP assays (Applied Biosystems, Foster City, CA, USA) were used for genotype analyses of the SNPs at the *IFNL3/4* locus. The TaqMan allele discrimination assays were carried out on an ABI ViiA 7 Real-time PCR System (Applied Biosystems) using probes labeled with fluorescent dyes (FAM and VIC) and nonfluorescent quencher according to the vendor’s instructions.

### Serum complement assay

Serum concentrations of complement C4 and C3 were determined by nephelometry. Complement depression was defined as the detection of both lower serum C4 (concentration < 100 mg/L) and C3 (concentration < 700 mg/L).

### Serum IFNL3 assay

An IFNL3 ELISA kit (catalog no. CSB-E13296h; CUSABIO, College Park, MD, USA) was used to measure serum IFNL levels of SLE patients according to the manufacturer’s instructions.

### Immunohistochemistry to detect IL-28B and IL-28 receptor in kidney tissue

The presence of IL-28B and expression of IL-28 receptor in kidney tissue were examined using kidney biopsies of lupus patients. Slides with the kidney biopsy sections were blocked with goat serum before being incubated with primary anti-IFNL3 antibodies (catalog no. A12908; ABclonal) and anti-IL-28 receptor alpha antibodies (catalog no. ab224395; Abcam) for 30 min at room temperature. The slides were washed three times with PBS before the addition of HRP-conjugated goat anti-mouse secondary antibodies. After extensive washing, DAB substrate was added to the slides for the detection of IFNL3 and IL-28 receptor.

### Statistical analysis

The Hardy–Weinberg equilibrium (HWE) was examined for all SNPs using chi-square tests. Three chi-square tests (the genotype test, the allele test, and the Cochran–Armitage trend test) were carried out with the SAS/Genetics software package release 8.2 (SAS Institute, Cary, NC, USA) to determine associations between individual SNPs and SLE susceptibility. To investigate the association between SNPs and SLE clinical manifestations, we stratified the clinical phenotypes according to SLE diagnosis criteria and assigned those SLE patients positive for a phenotype as “+” cases and assigned those negative as “–” cases. The allele and genotype distributions of SNPs between “+” cases and “–” cases were compared. The additive, dominant, and recessive models were used to analyze associations between SNP genotypes and phenotypes. To investigate the independent association between SLE clinical characteristics and SNP alleles/genotypes, multivariate logistic regressions were performed. The additive, dominant, and recessive allele effects for each SNP were modeled as the response variables and two categories of cases (“+” cases, “–” cases) were used as the independent variables pertaining to each clinical phenotype. In addition, logistic regressions adjusted for age and sex were used to calculate *P* values, odds ratios (ORs), and 95% confidence intervals (CIs) of risk alleles or genotypes. To account for multiple testing, Benjamini and Hochberg’s linear step-up method was carried out using the SAS MULTTEST procedure [19]. The false discovery rate (FDR)-adjusted *P* values are defined in a step-up fashion, with less conservative multipliers and control. A corrected *P* value (*P*_FDR_) less than 0.05 was considered statistically significant.

Linkage disequilibrium patterns of the *IFNL3/4* locus SNPs (Additional file 1: Figure S1) were analyzed by Haploview 4.2 (Broad Institute, Cambridge, MA, USA; http://www.broad.mit.edu/mpg/haploview). Haplotype information was inferred and frequencies were estimated using the HAPLOTYPE procedure of SAS 9.2 (SAS Institute). Haplotype frequency differences were then assessed between SLE cases and controls and between cases positive for and cases negative for a specific phenotype among SLE patients. To evaluate the independent association of each haplotype category, the permutation (*N* = 10,000) *P* values were calculated using the EM algorithm conditioned on the other haplotypes. Logistic regressions adjusted for sex and age were used to investigate the association between haplotype and SLE susceptibility and between cases positive for nephritis and cases negative for nephritis. Unpaired *t* tests were used to analyze the serum IFNL3 levels among SLE patients using GraphPad Prism 6.0 (GraphPad, La Jolla, CA, USA). *P* < 0.05 was considered significant.

## Results

### Characteristics of SLE patients

SLE patients (71 males and 760 females) and healthy controls (701 males and 919 females) were used in the genetic analyses of four SNPs (rs8099917, rs12979860, rs3682134815, and rs4803217) at the *IFNL3/4* locus (Additional file 1: Figure S1). The age onset of 831 SLE cases ranged from 8 to 77 years with an average age of 30.77 years (SD = 11.73) (Table 1). SLE cases consisted of 8.54% (71/831) males with an average age of 31.72 years (SD = 12.36) and 91.46% (760/831) females with an average age of 30.68 years (SD = 11.68). The ages of 1620 healthy controls ranged from 18 to 64 years and the average age of healthy controls was 41.22 years (SD = 10.47). The healthy controls consisted of 43.27% (701/1620) males with an average age of 40.26 years (SD = 9.26) and 56.73% (919/1620) females with an average age of 40.23 years (SD = 12.02). The clinical characteristics of the 831 SLE patients are presented in Table 1. Among the SLE patients, 55.48% (461/831) were positive for lupus nephritis (Table 1) according to the 1997 ACR diagnostic criteria either persistent proteinuria of greater than 0.5 g/d (or 3+ proteins on dipstick) or cellular casts of any type. For confirmation, another cohort of 182 SLE (100 with nephritis and 82 without nephritis) patients was used for lupus nephritis findings.

**Table 1:** Clinical characteristics of 831 Taiwanese SLE patients

	Count/available (%)	Male (*N* = 71)	Female (*N* = 760)
SLE case	831/831 (100.00%)	71/831 (8.54%)	760/831 (91.46%)
Age (years), mean ± standard deviation	30.77 ± 11.73	31.72 ± 12.36	30.68 ± 11.68
Oral ulcer	218/831 (26.23%)	14/71 (19.72%)	204/760 (26.84%)
Arthritis	522/831 (62.82%)	39/71 (54.93%)	483/760 (63.55%)
Malar rash	459/831 (55.23%)	39/71 (54.93%)	420/760 (55.26%)
Discoid rash	160/831 (19.25%)	18/71 (25.35%)	142/760 (18.68%)
Photosensitivity	187/831 (22.5%)	14/71 (19.72%)	173/760 (22.76%)
Pleural effusion	158/831 (19.01%)	12/71 (16.9%)	146/760 (19.21%)
Pericardial effusion	100/831 (12.03%)	13/71 (18.31%)	87/760 (11.45%)
Ascites	43/831 (5.17%)	3/71 (4.23%)	40/760 (5.26%)
Total counts for nephritis status	831 (100%)	71 (100%)	760 (100%)
Nephritis negative	370/831 (44.52%)	25/71 (35.21%)	345/760 (45.39%)
Nephritis positive	461/831 (55.48%)	46/71 (64.79%)	415/760 (54.61%)
Neuropsychiatric manifestations	133/831 (16%)	10/71 (14.08%)	123/760 (16.18%)
Leukopenia (WBC count < 3500/μl)	466/831 (56.08%)	41/71 (57.75%)	425/760 (55.92%)
Anemia (hemoglobin < 9 g/dl)	252/831 (30.32%)	11/71 (15.49%)	241/760 (31.71%)
Thrombocytopenia (platelet count < 10^5^/μl)	215/831 (25.87%)	23/71 (32.39%)	192/760 (25.26%)
Anti-dsDNA	618/813 (76.01%)	54/70 (77.14%)	564/743 (75.91%)
Complement depressed	632/818 (77.26%)	54/69 (78.26%)	578/749 (77.17%)
Anti-RNP	292/677 (43.13%)	24/62 (38.71%)	268/615 (43.58%)
Anti-Sm	256/678 (37.76%)	28/63 (44.44%)	228/615 (37.07%)
Anti-SSA	362/560 (64.64%)	30/48 (62.5%)	332/512 (64.84%)
Anti-SSB	149/560 (26.61%)	8/48 (16.67%)	141/512 (27.54%)
Anticardiolipin IgG	184/655 (28.09%)	12/50 (24%)	172/605 (28.43%)
Anticardiolipin IgM	55/600 (9.17%)	4/48 (8.33%)	51/552 (9.24%)

Data presented as count/available (%)

*SLE* systemic lupus erythematosus, *WBC* white blood cell

### Association of *IFNL3/4* SNPs with SLE susceptibility in patients negative for nephritis

Among four *IFNL4* SNPs, the distributions of three SNP genotypes were consistent with the Hardy–Weinberg equilibrium in both SLE patients and healthy controls. Only the *IFNL4* SNP ss469415590TT>ΔG (or rs3682134815) genotype distribution deviated from Hardy–Weinberg equilibrium, which is likely caused by the positive selection of the ss469415590TT allele favorable for humans fighting against viral infections [20–24]. We examined the single-locus association of four candidate SNPs in 831 SLE patients and 1620 healthy controls. As shown in Table 2, all four minor *IFNL3/4* SNP alleles (rs8099917G, rs12979860T, rs4803217A, and ss469415590ΔG) tended to associate with SLE susceptibility in the Cochran–Armitage trend test (rs8099917G, *P*_FDR_ = 0.009; rs12979860T, P_FDR_ = 0.0225; rs4803217A, *P*_FDR_ = 0.009; ss469415590ΔG, *P*_FDR_ = 0.0398). Nevertheless, the association between *IFNL3/4* SNPs and SLE susceptibility was not significant after adjustment for sex and age (*P*_FDR_ > 0.10). Subsequently, we analyzed the association between *IFNL3/4* SNPs and SLE susceptibility after stratifying SLE patients based on positivity of lupus nephritis. As shown in Table 2, all minor IFNL3/4 SNP alleles were significantly associated with SLE susceptibility in patients negative for nephritis compared to healthy controls adjusted for sex and age (rs8099917G, *P*_FDR_ = 0.00177, OR 1.68, 95% CI 1.24–2.28; rs12979860T, *P*_FDR_ = 0.00299, OR 1.58, 95% CI 1.18–2.32; rs4803217A, *P*_FDR_ = 0.00176, OR 1.65, 95% CI 1.22–2.23; and ss469415590ΔG, *P*_FD*R*_ = 0.00176, OR 1.70, 95% CI 1.26–2.29). In contrast, *IFN3/4* SNPs were not associated with SLE susceptibility in nephritis-positive patients (*P*_FDR_ > 0.9). Our data suggest that IFN-λ genetic variants may be a risk factor for the development of SLE in the subset of lupus nephritis-negative patients.

**Table 2:** Association of *IFNL3/4* SNPs with SLE susceptibility

SNP	Risk allele frequency	Genotype frequency			*P*_Trend_^*a*^	*P*_FDR_	Unadjusted^b^			Adjusted for sex and age^b^		
							*P*	*P*_FDR_	OR (95% CI)	*P*	*P*_FDR_	OR (95% CI)
rs8099917T > G	G	GG	GT	TT								
SLE	129 (7.76%)	3 (0.36%)	123 (14.8%)	705 (84.84%)	0.0035	0.009	0.0033	0.0084	1.42 (1.12–1.79)	0.1144	0.1144	1.26 (0.95–1.67)
Nephritis negative	75 (10.14%)	2 (0.54%)	71 (19.19%)	297 (80.27%)	0	0	8.57E–06	2.14E–05	1.90 (1.43–2.52)	0.00077	0.00177	1.68 (1.24–2.28)
Nephritis positive	54 (5.86%)	1 (0.22%)	52 (11.28%)	408 (88.50%)	0.7508	0.9401	0.74389	0.90622	1.05 (0.77–1.44)	0.91081	0.99622	1.02 (0.72–1.44)
Control	181 (5.57%)	8 (0.49%)	165 (10.16%)	1451 (89.35%)								
rs12979860C > T	T	CC	CT	TT								
SLE	133 (8%)	702 (84.48%)	125 (15.04%)	4 (0.48%)	0.0135	0.0225	0.0121	0.0202	1.34 (1.07–1.67)	0.0968	0.1144	1.26 (0.96–1.66)
Nephritis negative	76 (10.27%)	297 (80.27%)	70 (18.92%)	3 (0.81%)	0.0004	0.0005	7.19E–05	8.99E–05	1.75 (1.33–2.30)	0.00239	0.00299	1.58 (1.18–2.12)
Nephritis positive	57 (6.18%)	405 (87.85%)	55 (11.93%)	1 (0.22%)	0.9401	0.9401	0.89714	0.90622	1.02 (0.76–1.38)	0.87007	0.99622	1.03 (0.74–1.43)
Control	197 (6.07%)	1437 (88.49%)	177 (10.90%)	10 (0.62%)								
rs4803217C > A	A	AA	AC	CC								
SLE	131 (7.88%)	3 (0.36%)	125 (15.04%)	703 (84.6%)	0.0036	0.009	0.0029	0.0084	1.43 (1.13–1.8)	0.0386	0.1137	1.35 (1.02–1.8)
Nephritis negative	76 (10.27%)	2 (0.54%)	72 (19.46%)	296 (80.00%)	0	0	2.37E–05	3.96E–05	1.82 (1.38–2.41)	0.00106	0.00176	1.65 (1.22–2.23)
Nephritis positive	55 (5.97%)	1 (0.22%)	53 (11.50%)	407 (88.29%)	0.9368	0.9401	0.90622	0.90622	1.02 (0.75–1.38)	0.99622	0.99622	1.00 (0.71–1.40)
Control	190 (5.86%)	9 (0.56%)	172 (10.61%)	1440 (88.83%)								
ss469415590TT>ΔG^b^	ΔG	ΔG/ΔG	ΔG/TT	TT/TT								
SLE	142 (8.6%)	10 (1.21%)	122 (14.77%)	694 (84.02%)			0.0293	0.0367	1.26 (1.02–1.55)	0.0571	0.1137	1.28 (0.99–1.65)
Nephritis negative	76 (10.35%)	3(0.82%)	70 (19.07%)	294 (80.11%)	0	0	7.87E–06	2.14E–05	1.89 (1.43–2.50)	0.00058	0.00176	1.70 (1.26–2.29)
Nephritis positive	54 (5.88%)	1 (0.22%)	52 (11.33%)	406 (88.45%)	0.875	0.9401	0.83482	0.90622	1.03 (0.76–1.41)	0.98182	0.99622	1.00 (0.71–1.40)
Control	185 (5.70%)	8 (0.49%)	169 (10.41%)	1446 (89.09%)								

Data presented as *n* (%)

*SLE* systemic lupus erythematosus, *SNP* single-nucleotide polymorphism, *OR* odds ratio, *CI* confidence interval

^a^Trend test *P* values generated from 10,000 permutations

^b^Additive model used to test mode of inheritance

### Association of *IFNL3/4* SNPs with lupus nephritis

As a common phenotype, lupus nephritis represents a severe form of SLE. We subsequently analyzed whether *IFNL3/4* SNPs were associated with lupus nephritis among SLE patients. Table 3 shows that all major alleles of four *IFNL3/4* SNPs were significantly associated with the risk for nephritis (logistic regression analyses adjusted for sex and age: rs8099917T, *P*_FDR_ = 0.0021, OR 1.75, 95% CI 1.24–2.47; rs12979860C, *P*_FDR_ = 0.0034, OR 1.65, 95% CI 1.18–2.30; rs4803217C, *P*_FDR_ = 0.0021, OR 1.76, 95% CI 1.25–2.48; and ss469415590TT, *P*_FDR_ = 0.0021, OR 1.73, 95% CI 1.23–2.42). Our data show that the homozygosity of major alleles of four *IFNL3/4* SNPs is a major risk for lupus nephritis in SLE patients (Table 3). However, *IFNL3/4* SNPs were not significant associated with other manifestations such as arthritis, malar rash, leukopenia, positivity of anti-dsDNA/anti-RNP autoantibodies, and depressed complement levels among SLE patients (data not shown).

**Table 3:** Association of *IFNL3/4* SNPs with lupus nephritis among SLE patients

SNP	Risk allele frequency	Genotype frequency			*P*_Trend_^a^	*P*_FDR_	Test for mode of inheritance unadjusted				Test for mode of inheritance adjusted for sex and age			
								*P*	*P*_FDR_	OR (95% CI)		*P*	*P*_FDR_	OR (95% CI)
rs8099917T > G	T	GG	GT	TT			Additive	0.0018	0.0024	1.73 (1.23–2.45)	Additive	0.0016	0.0021	1.75 (1.24–2.47)
Nephritis^+^	1058 (94.30%)	1 (0.18%)	62 (11.05%)	498 (88.77%)	0.001	0.0016	TT + GT vs GG	0.45681	0.657	2.49 (0.23–27.60)	TT vs GT + GG	0.4705	0.684	2.42 (0.22–26.82)
Cohort 1	868 (94.14%)	1 (0.22%)	52 (11.28%)	408 (88.5%)										
Cohort 2	190 (95.00%)	0 (0.00%)	10 (10.00%)	90 (90.00%)										
Nephritis^−^	818 (90.69%)	2 (0.44%)	80 (17.74%)	369 (81.82%)			TT vs GT + GG	0.0019	0.0025	1.76 (1.23–2.50)	TT + GT vs GG	0.0016	0.0022	1.77 (1.24–2.53)
Cohort 1	665 (89.86%)	2 (0.54%)	71 (19.19%)	297 (80.27%)										
Cohort 2	153 (94.44%)	0 (0.00%)	9 (11.11%)	72 (88.89%)										
rs12979860T > C	C	CC	CT	TT			Additive	0.0036	0.0036	1.64 (1.18–2.29)	Additive	0.0034	0.0034	1.65 (1.18–2.30)
Nephritis^+^	1053 (93.85%)	494 (88.06%)	65 (11.59%)	2 (0.36%)	0.0042	0.0042	CC + CT vs TT	0.4949	0.4949	1.87 (0.31–11.22)	CC vs CT + TT	0.5153	0.5153	1.81 (0.30–10.91)
Cohort 1	865 (93.82%)	405 (87.85%)	55 (11.93%)	1 (0.22%)										
Cohort 2	188 (94.00%)	89 (89.00%)	10 (10.00%)	1 (1.00%)										
Nephritis^−^	817 (90.38%)	368 (81.42%)	81 (17.92%)	3 (0.66%)			CC vs CT + TT	0.0034	0.0136	1.68(1.19–2.38)	CC + CT vs TT	0.0031	0.0123	1.69 (1.19–2.40)
Cohort 1	664 (89.73%)	297 (80.27%)	70 (18.92%)	3 (0.81%)										
Cohort 2	153 (93.29%)	71 (86.59%)	11 (13.41%)	0 (0.00%)										
rs4803217C > A	C	AA	AC	CC			Additive	0.0012	0.0024	1.76(1.25–2.47)	Additive	0.0011	0.0021	1.76 (1.25–2.48)
Nephritis^+^	1055 (94.20%)	2 (0.36%)	61 (10.89%)	497 (88.75%)	0.0012	0.0016	CC + AC vs AA	0.8300	0.83	1.24 (0.17–8.84)	CC vs AC + AA	0.8531	0.8531	1.20 (0.17–8.58)
Cohort 1	867 (94.03%)	1 (0.22%)	53 (11.5%)	407 (88.29%)										
Cohort 2	188 (94.95%)	1 (1.01%)	8 (8.08%)	90 (90.91%)										
Nephritis^−^	817 (90.38%)	2 (0.44%)	83 (18.36%)	367 (81.19%)			CC vs AC + AA	0.0008	0.0025	1.83 (1.28–2.60)	CC + AC vs AA	0.0007	0.0022	1.84 (1.29–2.61)
Cohort 1	664 (89.73%)	2 (0.54%)	72 (19.46%)	296 (80%)										
Cohort 2	153 (93.29%)	0 (0.00%)	11 (13.41%)	71 (86.59%)										
ss469415590TT>ΔG^b^	TT	ΔG/ΔG	ΔG/TT	TT/TT			Additive	0.0015	0.0024	1.72 (1.23–2.42)	Additive	0.0015	0.0021	1.73(1.23–2.42)
Nephritis^+^	1052 (94.10%)	2 (0.36%)	62 (11.09%)	495 (88.55%)	0.0011	0.0016	BB + AB vs AA	0.4927	0.657	1.87 (0.31–11.26)	2TT vs GTT + GG	0.513	0.684	1.82 (0.30–10.94)
Cohort 1	858 (93.46%)	4 (0.87%)	52 (11.33%)	403 (87.8%)										
Cohort 2	188 (94.00%)	1 (1.00%)	10 (10.00%)	89 (89.00%)										
Nephritis^−^	811 (90.31%)	3 (0.67%)	81 (18.04%)	365 (81.29%)			BB vs AB + AA	0.0013	0.0025	1.78 (1.25–2.53)	2TT + GTT vs GG	0.0012	0.0022	1.79 (1.26–2.54)
Cohort 1	652 (88.83%)	6 (1.63%)	70 (19.07%)	291 (79.29%)										
Cohort 2	153 (93.29%)	0 (0.00%)	11 (13.41%)	71 (86.59%)										

Data presented as *n* (%)

*SLE* systemic lupus erythematosus, *SNP* single-nucleotide polymorphism, *OR* odds ratio, *CI* confidence interval

^a^Trend test *P* values generated from 10,000 permutations

^b^Genotypes of ΔG/ΔG, ΔG/TT, and TT/TT are also named GG, GTT, and 2TT, respectively

### Association of *IFNL3/4* SNP haplotypes with lupus nephritis

*IFNL3/4* SNPs are in strong linkage disequilibrium (Additional file 1: Figure S2). Subsequently, we used haplotype analysis to determine whether *IFNL3/4* SNP haplotypes (rs8099917, ss469415590, rs12979860, and rs4803217) are associated with the risk for nephritis among SLE patients. As shown in Table 4, the most common haplotype (T-TTC-C) was significantly associated with the risk for lupus nephritis (logistic regression adjusted for sex and age: *P* = 0.0015, OR 1.68, 95% CI 1.22–2.32) while the minor haplotype (G-ΔG-T-A) was associated with the low risk for lupus nephritis (adjusted *P* = 0.0011, OR 0.50, 95% CI 0.33–0.76). A combination of two cohorts of SLE patients revealed similar significant findings (Additional file 1: Table S1). However, *IFNL3/4* SNP haplotypes were not associated with other manifestations including oral ulcer, arthritis, malar rash, discoid rash, photosensitivity, pleural effusion, pericardial effusion, ascites, neuropsychiatric manifestations, leukopenia, anemia, thrombocytopenia, anti-dsDNA, complement depressed, anti-RNP, anti-Sm, anti-SSA, anti-SSB, anticardiolipin IgG, and anticardiolipin IgM) when compared among SLE patients (data not shown). Our data suggest that IFN-λs have a unique role in the development of lupus nephritis.

**Table 4:** Association of *IFN3/4* locus SNP haplotypes (rs8099917-ss469415590-rs12979860-rs4803217) with lupus nephritis among SLE patients

Haplotype	Estimated frequency (%)			Permutation	Logistic regression		Logistic regression adjusted for sex and age	
	Nephritis^+^	Nephritis^−^	SLE cases	P*	*P*	OR (95% CI)	*P*	OR (95% CI)
	(*N* = 461)	(*N* = 370)	(*N* = 831)					
T-TT-C-C	91.74	86.99	89.62	0.0024	0.0018	1.66 (1.21–2.28)	0.0015	1.68 (1.22–2.32)
G-ΔG-T-A	4.44	8.23	6.13	0.0019	0.0016	0.52 (0.34–0.78)	0.0011	0.5 (0.33–0.76)
Others	3.83	4.78	4.25		0.3472	0.79 (0.49–1.28)	0.3690	0.8 (0.49–1.3)

Data presented as *n* (%)

*The *p*-values for the estimated haplotype were generated from 10,000 permutations using the EM algorithm

*SLE* systemic lupus erythematosus, *SNP* single-nucleotide polymorphism, *OR* odds ratio, *CI* confidence interval

### IFNL-λ3 (IFNL3) levels correlated with SLE disease activity and complement depression

**Fig. 1:**
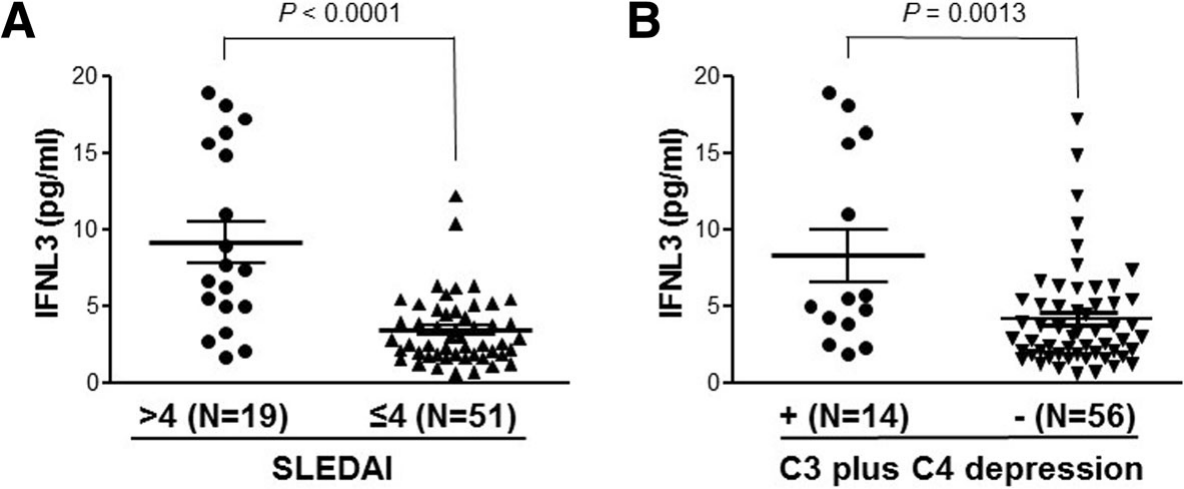
Association of serum IFNL3 levels with SLEDAI and complement depression. **a** IFNL3 levels significantly (unpaired *t* test *t* = 5.974, *P* < 0.0001) increased in high SLEDAI SLE patients (SLEDAI > 4, *N* = 19; IFNL3 concentration 9.190 ± 1.351 pg/ml) compared to low SLEDAI SLE patients (SLEDAI ≤ 4, *N* = 51; IFNL3 concentration 3.413 ± 0.3171 pg/ml). **b** IFNL3 levels also significantly (unpaired *t* test *t* = 3.362, *P* = 0.0013) higher in SLE patients (*N* = 14) with complement C3 plus C4 depression (IFNL3 concentration 8.288 ± 1.696 pg/ml) than in those (*N* = 56) without complement C3 plus C4 depression (IFNL3 concentration 4.154 ± 0.4514 pg/ml). IFNL3 interferon-λ3, SLEDAI Systemic Lupus Erythematosus Disease Activity Index

We subsequently performed correlation analyses of serum IFNL3 levels with traditional clinical and laboratory parameters. As shown in Fig. 1, we found that the serum IFNL3 levels were significantly increased in SLE patients with high SLE disease activity index (SLEDAI > 4, *N* = 19; IFNL3 concentration 9.190 ± 1.351 pg/ml) as compared to the patients with low disease activity (SLEDAI ≤ 4, *N* = 51; IFNL3 concentration 3.413 ± 0.3171 pg/ml) (*P* < 0.0001). In addition, SLE patients with both depressed C3 and C4 had significantly higher serum IFNL3 (*N* = 14) than those without complement C3 plus C4 depression (*N* = 56) (IFNL3 concentration 8.288 ± 1.696 pg/ml vs 4.154 ± 0.4514 pg/ml; *P* = 0.0013). We confirmed that IFNL3 levels were significantly associated with SLEDAI in an independent cohort (Additional file 1: Figure S3A). However, IFNL3 levels were not significantly different (unpaired *t* test *t* = 1.650, *P* = 0.103) between nephritis-positive patients and nephritis-negative patients (Additional file 1: Figure S3B). Our data suggest that serum IFNL3 could be used as a disease activity biomarker for SLE.

### Detection of IL-28B and IL-28 receptors in kidney tissue of SLE patients

**Fig. 2:**
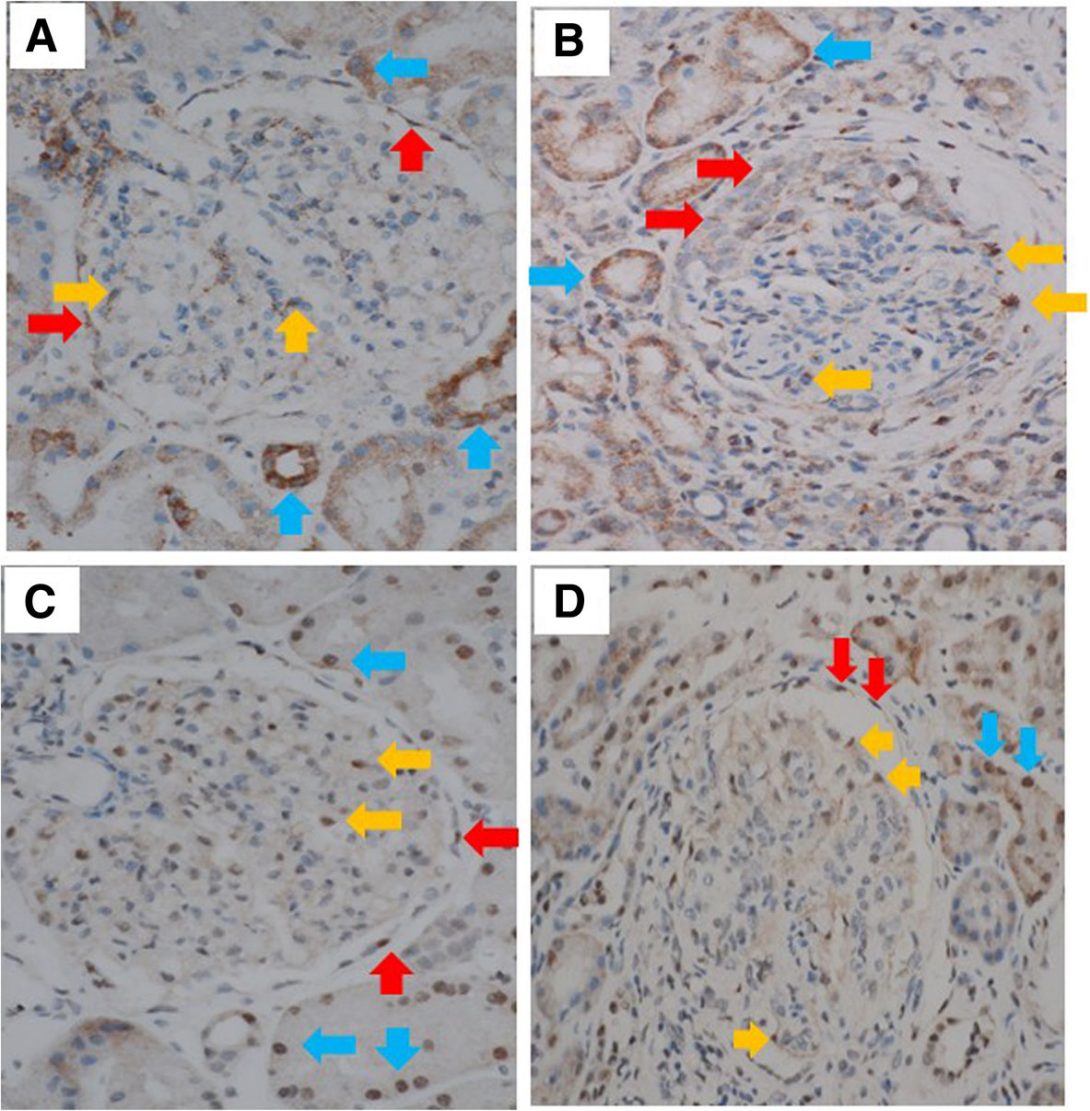
Detection of IL-28B (IFNL3) and IL-28 receptor alpha (IL-28RA) in kidney tissue of lupus patients with nephritis. IFNL3 detected on parietal cells (red arrow), podocytes (yellow arrows), and tubular cells (blue arrows) of kidney from lupus patient with minimal change disease (**a**) and lupus patient with class IV proliferative nephritis (**b**). IL-28RA expressed in parietal cells (red arrow), podocytes (yellow arrows), and tubular cells (blue arrows) of kidney from patient with minimal change disease (**c**) and lupus patient with class IV proliferative nephritis (**d**)

Since *IFNL3/4* SNP haplotypes were associated with lupus nephritis, we carried out immunohistochemistry analyses to examine the presence of IFNL and its receptor in kidney tissues of three SLE patients with nephritis. IFNL3 were detected on parietal cells (red arrow), podocytes (yellow arrows), and tubular cells (blue arrows) (Fig. 2a, b), which expressed IL-28 receptor alpha (IL-28RA) (Fig. 2c, d). Our data support the concept that kidney tissue is a target of IFNLs.

## Discussion

IFN-λs (IFNLs) play critical roles in innate and adaptive immune responses [4]. Recent genetic studies revealed that IFN-λ genes contribute to the spontaneous resolution of HCV and that IFN-λ genetic variants are reliable biomarkers for treatment outcomes of HCV infections [5]. SLE is a heterogeneous disease with varied clinical phenotypes. In the current study, we demonstrated that *IFNL3/4* genetic variants were significantly associated with SLE susceptibility in lupus nephritis-negative patients. Specifically, minor alleles of all *IFNL3/4* SNPs are risk factors for SLE development in patients without nephritis. In contrast, the major alleles of *IFNL3/4* SNPs are a significant risk factor for the development of nephritis among SLE patients. Our study is the first to reveal that IFN-λ genes play a unique role in the development of SLE and lupus nephritis, indicating that IFN-λ genetic variants could be potential biomarkers for SLE susceptibility and lupus nephritis.

Type I IFNs contribute to the breakdown of immune tolerance by enhancing the differentiation of immature myeloid dendritic cells (mDCs) into mature DCs that drive the expansion and differentiation of autoreactive T cells and B cells. Type I IFN-matured DCs also activate cytotoxic CD8^+^ T cells that kill susceptible target cells. Type I IFNs are key cytokines in the pathogenesis of SLE [25]. Mouse models confirmed that type I IFNs accelerate disease progression through the increase of autoantibody production and the development of nephritis [26, 27]. IFN-λs (IFN-λ1, IFN-λ2, IFN-λ3, and IFN-λ4) are structurally related to the IL-10 family that transduces cellular signals through a heterodimeric IFN-λ receptor complex composed of a unique IL28RA/IFN-λR1 (IFN-λ-specific ligand binding chain) and a shared IL-10Rβ chain (a subunit of the receptors for IL-10, IL-22, and IL-26) [6, 28, 29]. The binding of IFN-λs to the IFN-λ-receptor complex activates the Janus kinase–signal transducer and activator of transcription (Jak–STAT) pathway, leading to the expressions of IFN-regulated genes (ISGs) that inhibit viral replication [4, 28, 30, 31].

IFN-λ-stimulated DCs express high levels of MHC class I and MHC class II but low levels of costimulatory molecules. IFN-λ-exposed DCs specifically induce IL-2-dependent proliferation of CD4^+^CD25^+^Foxp3^+^ suppressive T cells that inhibit the T-cell proliferation driven by mature DCs. Therefore, IFN-λs favor the generation of tolerogenic DCs that thwart type I IFN functions [32]. Interestingly, as an important target of IFN-λs, neutrophils also express high levels of IL28RA/IFN-λR1. IFN-λs inhibit neutrophil recruitment and activation, preventing the amplification of inflammation. Furthermore, IFN-λs could completely halt and reverse the development of collagen-induced arthritis [33]. As a key regulator to inhibit B-cell immune responses, IFN-λ3 treatment dramatically reduced antigen-stimulated B-cell proliferation and IgG production through suppressing Th2 cytokine production [34]. Taken together, IFN-λs appear to inhibit chronic inflammation through the actions of DCs, suppressive T cells, neutrophils, and B cells [33].

*IFNL3/4* locus SNPs are strongly associated with clearance of HCV [35–42]. *IFNL3* 3′-untranslated region (UTR) SNP rs4803217 significantly influences AU-rich element-mediated *IFNL3* mRNA decay. *IFNL3* mRNA containing the minor rs4803217A allele is much less stable than that with the major rs4803217C allele. Therefore, the major rs4803217C allele is a high IFN-λ3 producer while the minor rs4803217A allele is a low IFN-λ3 producer [43]. It is reasonable to assume that the most common *IFNL3/4* SNP haplotype rs8099917T/ss469415590/rs12979860C/rs4803217C containing the rs4803217C allele is a high producer of IFN-λ3, which is assumed to suppress the development of autoimmune inflammation [33]. We found that the most common *IFNL3/4* SNP haplotype containing the rs4803217C allele was significantly associated with the low risk for SLE in nephritis-negative patients, confirming that a high producer of IFN-λ3 may have a protective role against SLE.

Notably, the newly identified *IFNL4* SNP ss469415590 TT>ΔG alters the *IFNL4* reading frame and the rs368234815ΔG allele results in the open reading frame *IFNL4* mRNA. Nevertheless, IFN-λ4 peptide produced from the *IFNL4* ss469415590ΔG allele is a dysfunctional cytokine [22], which may explain the defective HCV clearance in Africans, Europeans, and Asians with the *IFNL4* ss469415590ΔG allele [20, 22–24, 44, 45]. On the other hand, the major ss469415590TT allele with a disrupted *IFNL4* open reading frame is associated with the increased expression of IFN-λ3 [21, 23, 24]. Our study revealed that the minor rs3682134815ΔG allele carrier is also a risk for SLE susceptibility in the subset of SLE patients negative for lupus nephritis, indicating that the expression of dysfunctional IFN-λ4 in combination with the low IFN-λ3 production has a role in the pathogenesis of SLE. IFN-λ3 levels have been linked to SLE disease activity, complement, and autoantibody (anti-Ro/SSA) status [46]. In the current study, we found that high levels of IFN-λ3 were significantly associated with high SLEDAI and complement depression. The increased production of IFN-λ3 in SLE patients with a high SLEDAI may reflect an intrinsic mechanism to suppress chronic inflammation. IFN-λ3 levels may be a useful biomarker for SLE disease activity.

Paradoxically, our study revealed that the most common *IFNL3/4* SNP haplotype rs8099917T/rs12979860C/rs4803217C (high IFN-λ3 producer) was significantly associated with the risk for lupus nephritis, while the minor haplotype rs8099917G/rs12979860T/rs4803217A (low IFN-λ3 producer) had a protective role against lupus nephritis. We speculate several possible explanations. First, IFN-λs possess the highest cytotoxic potential as they induce more robust cell death than type I IFNs and type II IFNs [47]. Kidney cells express the IFN-λ3 receptor and could be very susceptible to IFN-λ-induced apoptosis, leading to necrotic inflammation and kidney injury. Indeed, we have detected both IFN-λ3 (IFNL3) and IL-28 receptor alpha in kidney tissue, suggesting a pathogenic mechanism of IFN-λ3 in the development of SLE nephritis. Second, the high levels of proinflammatory cytokines such as type I IFNs and IL-6 in SLE patients may reverse the anti-inflammatory action of IFN-λs, which subsequently exacerbates kidney injury under the circumstances of inflammation. Indeed, in patients with chronic hepatitis C (CHC), while the favorable genotypes responsible for high levels of IFN-λ production increase viral clearance, patients with the high IFN-λ-producer genotypes were twice as likely to develop adverse clinical outcomes [48, 49]. Finally, the *IFNL3/4* risk SNP haplotype may be in linkage disequilibrium with unidentified causative SNPs and/or may interact with other genes to cause lupus nephritis. Nevertheless, the *IFNL3/4* locus at chromosome 19q13 has never been identified to contain risk gene(s) for SLE susceptibility by GWAS [14, 15]. The absence of association of *IFNL3/4* SNPs with SLE in previous studies could be explained by our observation that the *IFNL3/4* SNPs are a risk factor for SLE susceptibility in the subset of lupus nephritis-negative patients. Further mechanistic studies are needed to pinpoint the precise role of IFN-λs in the development of lupus nephritis.

Nevertheless, the current study has several limitations. First, the cross-sectional serum IFNL3 levels were determined in a modest number of SLE patients. Studies with large clinical samples and longitudinal data are required to establish the association between serum IFNL3 levels and SLE disease activity. Second, since IFNL3 production could be affected by disease activity, a large number of SLE patients in quiescent disease status need to be used to determine the effect of *IFNL3/4* SNPs on IFNL3 production. Finally, extensive in-vivo and in-vitro studies are required to delineate the mechanistic roles of IFNLs in SLE development and lupus nephritis.

## Conclusions

*IFNL3/4* SNPs are significantly associated with SLE susceptibility and lupus nephritis in Taiwanese. High levels of IFN-λs may have a protective role against the development of SLE in the initial stage, but the increased and persistent production of IFN-λs may predispose SLE patients to the development of lupus nephritis. Our data point to a distinctive role of IFN-λs in the development of autoimmune diseases and phenotypes. IFN-λs may be a potential therapeutic target in treating lupus nephritis.

## Additional file

Additional file 1: **Table S1.** Association of *IFN3/4* locus SNP haplotypes (rs8099917-ss469415590-rs12979860-rs4803217) with lupus nephritis among SLE patients. **Figure S1.** Schematic illustration of *IFNL3/4* locus SNP locations. Sizes of exons and distances between exons indicated as base pairs (bp). First exon (exon 1) of each gene starts from ATG start codon and last exon (exon 5) of each gene ends at stop codon. SNP rs8099917 located in *IFNL4* promoter region (3945 bp upstream of translation starting site) and SNP rs4803217 in *IFNL3* 3′-UTR (52 bp downstream of translation termination codon). Two other SNPs (ss469415590 and rs12979860) are with *IFNL4* gene. **Figure S2.** Pairwise LD patterns of four *IFNL3/4* locus SNPs on chromosome 19 show coefficient of linkage disequilibrium *D*′ (red) and square of correlation coefficient between two indicator variables γ^2^ (black) of all subjects (**A**), SLE cases (**B**), and healthy controls (**C**), respectively. Darker colors indicate stronger LD. **Figure S3.** Association of IFN3 levels with SLE disease activity (SLEDAI) in replication cohort. **A** IFNL3 levels significantly (unpaired *t* test *t* = 3.783, *P* = 0.0003) increased in high SLEDAI SLE patients (SLEDAI ≥ 4, *N* = 40; IFNL3 concentration 8.450 ± 1.263 pg/ml) than in low SLEDAI patients (SLEDAI = 0, *N* = 40; IFNL3 concentration 3.260 ± 0.5365 pg/ml). **B** IFNL3 levels not significantly different (unpaired *t* test *t* = 1.650, *P* = 0.103) between nephritis-positive patients (*N* = 40; IFNL3 concentration 4.645 ± 1.039 pg/ml) and nephritis-negative patients (*N* = 40; IFNL3 concentration 7.065 ± 1.036 pg/ml). (DOCX 194 kb)

## Abbreviations

CHC: Chronic hepatitis C
HWE: Hardy–Weinberg equilibrium
IFN: Interferon
ISG: IFN-regulated gene
JAK: Janus kinase
mDC: Myeloid dendritic cell
SLE: Systemic lupus erythematosus
SLEDAI: Systemic Lupus Erythematosus Disease Activity Index
SNP: Single-nucleotide polymorphism
STAT: Signal transducer and activator of transcription
UTR: Untranslated region

## References

[1] Tsokos GC (2011). Systemic lupus erythematosus. N Engl J Med.

[2] Azevedo PC, Murphy G, Isenberg DA (2014). Pathology of systemic lupus erythematosus: the challenges ahead. Methods Mol Biol.

[3] Kow NY, Mak A (2013). Costimulatory pathways: physiology and potential therapeutic manipulation in systemic lupus erythematosus. Clin Dev Immunol.

[4] Galani IE, Koltsida O, Andreakos E (2015). Type III interferons (IFNs): emerging master regulators of immunity. Adv Exp Med Biol.

[5] Kelly C, Klenerman P, Barnes E (2011). Interferon lambdas: the next cytokine storm. Gut.

[6] Zdanov A (2010). Structural analysis of cytokines comprising the IL-10 family. Cytokine Growth Factor Rev.

[7] Sozzani S, Bosisio D, Scarsi M, Tincani A (2010). Type I interferons in systemic autoimmunity. Autoimmunity.

[8] Ronnblom L, Alm GV, Eloranta ML (2011). The type I interferon system in the development of lupus. Semin Immunol.

[9] Ronnblom L, Eloranta ML (2013). The interferon signature in autoimmune diseases. Curr Opin Rheumatol.

[10] Obermoser G, Pascual V (2010). The interferon-alpha signature of systemic lupus erythematosus. Lupus.

[11] Bennett L, Palucka AK, Arce E, Cantrell V, Borvak J, Banchereau J, Pascual V (2003). Interferon and granulopoiesis signatures in systemic lupus erythematosus blood. J Exp Med.

[12] Bronson PG, Chaivorapol C, Ortmann W, Behrens TW, Graham RR (2012). The genetics of type I interferon in systemic lupus erythematosus. Curr Opin Immunol.

[13] Sandling JK, Garnier S, Sigurdsson S, Wang C, Nordmark G, Gunnarsson I, Svenungsson E, Padyukov L, Sturfelt G, Jonsen A (2011). A candidate gene study of the type I interferon pathway implicates IKBKE and IL8 as risk loci for SLE. Eur J Hum Genet.

[14] Morris DL, Sheng Y, Zhang Y, Wang YF, Zhu Z, Tombleson P, Chen L, Cunninghame Graham DS, Bentham J, Roberts AL (2016). Genome-wide association meta-analysis in Chinese and European individuals identifies ten new loci associated with systemic lupus erythematosus. Nat Genet.

[15] Teruel M, Alarcon-Riquelme ME (2016). The genetic basis of systemic lupus erythematosus: what are the risk factors and what have we learned. J Autoimmun.

[16] Boisvert M, Shoukry NH (2016). Type III interferons in hepatitis C virus infection. Front Immunol.

[17] Hochberg MC (1997). Updating the American College of Rheumatology revised criteria for the classification of systemic lupus erythematosus. Arthritis Rheum.

[18] Bombardier C, Gladman DD, Urowitz MB, Caron D, Chang CH (1992). Derivation of the SLEDAI. A disease activity index for lupus patients. The committee on prognosis studies in SLE. Arthritis Rheum.

[19] Benjamini Y, Hochberg Y. Controlling the false discovery rate: a practical and powerful approach to multiple testing. J R Stat Soc Ser B Methodol. 1995;57:289–300.

[20] Aka PV, Kuniholm MH, Pfeiffer RM, Wang AS, Tang W, Chen S, Astemborski J, Plankey M, Villacres MC, Peters MG (2014). Association of the IFNL4-DeltaG allele with impaired spontaneous clearance of hepatitis C virus. J Infect Dis.

[21] Bibert S, Roger T, Calandra T, Bochud M, Cerny A, Semmo N, Duong FH, Gerlach T, Malinverni R, Moradpour D (2013). IL28B expression depends on a novel TT/−G polymorphism which improves HCV clearance prediction. J Exp Med.

[22] Hamming OJ, Terczynska-Dyla E, Vieyres G, Dijkman R, Jorgensen SE, Akhtar H, Siupka P, Pietschmann T, Thiel V, Hartmann R (2013). Interferon lambda 4 signals via the IFNlambda receptor to regulate antiviral activity against HCV and coronaviruses. EMBO J.

[23] Prokunina-Olsson L, Muchmore B, Tang W, Pfeiffer RM, Park H, Dickensheets H, Hergott D, Porter-Gill P, Mumy A, Kohaar I (2013). A variant upstream of IFNL3 (IL28B) creating a new interferon gene IFNL4 is associated with impaired clearance of hepatitis C virus. Nat Genet.

[24] Key FM, Peter B, Dennis MY, Huerta-Sanchez E, Tang W, Prokunina-Olsson L, Nielsen R, Andres AM (2014). Selection on a variant associated with improved viral clearance drives local, adaptive pseudogenization of interferon lambda 4 (IFNL4). PLoS Genet.

[25] Banchereau J, Pascual V (2006). Type I interferon in systemic lupus erythematosus and other autoimmune diseases. Immunity.

[26] Liu Z, Bethunaickan R, Huang W, Lodhi U, Solano I, Madaio MP, Davidson A (2011). Interferon-alpha accelerates murine systemic lupus erythematosus in a T cell-dependent manner. Arthritis Rheum.

[27] Liu Z, Bethunaickan R, Huang W, Ramanujam M, Madaio MP, Davidson A (2011). IFN-alpha confers resistance of systemic lupus erythematosus nephritis to therapy in NZB/W F1 mice. J Immunol.

[28] Gad HH, Hamming OJ, Hartmann R (2010). The structure of human interferon lambda and what it has taught us. J Interf Cytokine Res.

[29] Commins S, Steinke JW, Borish L (2008). The extended IL-10 superfamily: IL-10, IL-19, IL-20, IL-22, IL-24, IL-26, IL-28, and IL-29. J Allergy Clin Immunol.

[30] Gad HH, Dellgren C, Hamming OJ, Vends S, Paludan SR, Hartmann R (2009). Interferon-lambda is functionally an interferon but structurally related to the interleukin-10 family. J Biol Chem.

[31] Zheng YW, Li H, Yu JP, Zhao H, Wang SE, Ren XB (2013). Interferon-lambdas: special immunomodulatory agents and potential therapeutic targets. J Innate Immun.

[32] Mennechet FJ, Uze G (2006). Interferon-lambda-treated dendritic cells specifically induce proliferation of FOXP3-expressing suppressor T cells. Blood.

[33] Blazek K, Eames HL, Weiss M, Byrne AJ, Perocheau D, Pease JE, Doyle S, McCann F, Williams RO, Udalova IA (2015). IFN-lambda resolves inflammation via suppression of neutrophil infiltration and IL-1beta production. J Exp Med.

[34] Egli A, Santer DM, O'Shea D, Barakat K, Syedbasha M, Vollmer M, Baluch A, Bhat R, Groenendyk J, Joyce MA (2014). IL-28B is a key regulator of B- and T-cell vaccine responses against influenza. PLoS Pathog.

[35] Ge D, Fellay J, Thompson AJ, Simon JS, Shianna KV, Urban TJ, Heinzen EL, Qiu P, Bertelsen AH, Muir AJ (2009). Genetic variation in IL28B predicts hepatitis C treatment-induced viral clearance. Nature.

[36] Rauch A, Kutalik Z, Descombes P, Cai T, Di Iulio J, Mueller T, Bochud M, Battegay M, Bernasconi E, Borovicka J (2010). Genetic variation in IL28B is associated with chronic hepatitis C and treatment failure: a genome-wide association study. Gastroenterology.

[37] Suppiah V, Moldovan M, Ahlenstiel G, Berg T, Weltman M, Abate ML, Bassendine M, Spengler U, Dore GJ, Powell E (2009). IL28B is associated with response to chronic hepatitis C interferon-alpha and ribavirin therapy. Nat Genet.

[38] Tanaka Y, Nishida N, Sugiyama M, Kurosaki M, Matsuura K, Sakamoto N, Nakagawa M, Korenaga M, Hino K, Hige S (2009). Genome-wide association of IL28B with response to pegylated interferon-alpha and ribavirin therapy for chronic hepatitis C. Nat Genet.

[39] Thomas DL, Thio CL, Martin MP, Qi Y, Ge D, O'Huigin C, Kidd J, Kidd K, Khakoo SI, Alexander G (2009). Genetic variation in IL28B and spontaneous clearance of hepatitis C virus. Nature.

[40] Duggal P, Thio CL, Wojcik GL, Goedert JJ, Mangia A, Latanich R, Kim AY, Lauer GM, Chung RT, Peters MG (2013). Genome-wide association study of spontaneous resolution of hepatitis C virus infection: data from multiple cohorts. Ann Intern Med.

[41] Bota S, Sporea I, Sirli R, Neghina AM, Popescu A, Strain M (2013). Role of interleukin-28B polymorphism as a predictor of sustained virological response in patients with chronic hepatitis C treated with triple therapy: a systematic review and meta-analysis. Clin Drug Investig.

[42] Shakado S, Sakisaka S, Okanoue T, Chayama K, Izumi N, Toyoda J, Tanaka E, Ido A, Takehara T, Yoshioka K, et al. Interleukin 28B polymorphism predicts interferon plus ribavirin treatment outcome in patients with hepatitis C virus-related liver cirrhosis: a multicenter retrospective study in Japan. Hepatol Res. 2014;44:983–92. 10.1111/hepr.12280 24400682

[43] McFarland AP, Horner SM, Jarret A, Joslyn RC, Bindewald E, Shapiro BA, Delker DA, Hagedorn CH, Carrington M, Gale M (2014). The favorable IFNL3 genotype escapes mRNA decay mediated by AU-rich elements and hepatitis C virus-induced microRNAs. Nat Immunol.

[44] Fujino H, Imamura M, Nagaoki Y, Kawakami Y, Abe H, Hayes CN, Kan H, Fukuhara T, Kobayashi T, Masaki K (2014). Predictive value of the IFNL4 polymorphism on outcome of telaprevir, peginterferon, and ribavirin therapy for older patients with genotype 1b chronic hepatitis C. J Gastroenterol.

[45] Lu YF, Goldstein DB, Urban TJ, Bradrick SS (2015). Interferon-lambda4 is a cell-autonomous type III interferon associated with pre-treatment hepatitis C virus burden. Virology.

[46] Amezcua-Guerra LM, Marquez-Velasco R, Chavez-Rueda AK, Castillo-Martinez D, Masso F, Paez A, Colin-Fuentes J, Bojalil R (2017). Type III interferons in systemic lupus erythematosus: association between interferon lambda3, disease activity, and anti-Ro/SSA antibodies. J Clin Rheumatol.

[47] Li W, Lewis-Antes A, Huang J, Balan M, Kotenko SV (2008). Regulation of apoptosis by type III interferons. Cell Prolif.

[48] Noureddin M, Wright EC, Alter HJ, Clark S, Thomas E, Chen R, Zhao X, Conry-Cantilena C, Kleiner DE, Liang TJ (2013). Association of IL28B genotype with fibrosis progression and clinical outcomes in patients with chronic hepatitis C: a longitudinal analysis. Hepatology.

[49] Petta S, Grimaudo S, Camma C, Cabibi D, Di Marco V, Licata G, Pipitone RM, Craxi A (2012). IL28B and PNPLA3 polymorphisms affect histological liver damage in patients with non-alcoholic fatty liver disease. J Hepatol.

