# Supplementary material for: Interferon-λ3/4 genetic variants and interferon-λ3 serum levels are biomarkers of lupus nephritis and disease activity in Taiwanese
Source: Arthritis Res Ther. 2018 Aug 29;20:193. doi: 10.1186/s13075-018-1683-z (PMC6116434; doi:10.1186/s13075-018-1683-z)
Supplement: Supplementary file 1 — Table S1. Association of IFN3/4 locus SNP haplotypes (rs8099917-ss469415590-rs12979860-rs4803217) with lupus nephritis among SLE patients. Figure S1. Schematic illustration of IFNL3/4 locus SNP locations. Sizes of exons and distances between exons indicated as base pairs (bp). First exon (exon 1) of each gene starts from ATG start codon and last exon (exon 5) of each gene ends at stop codon. SNP rs8099917 located in IFNL4 promoter region (3945 bp upstream of translation starting site) and SNP rs4803217 in IFNL3 3′-UTR (52 bp downstream of translation termination codon). Two other SNPs (ss469415590 and rs12979860) are with IFNL4 gene. Figure S2. Pairwise LD patterns of four IFNL3/4 locus SNPs on chromosome 19 show coefficient of linkage disequilibrium D′ (red) and square of correlation coefficient between two indicator variables γ2 (black) of all subjects (A), SLE cases (B), and healthy controls (C), respectively. Darker colors indicate stronger LD. Figure S3. Association of IFN3 levels with SLE disease activity (SLEDAI) in replication cohort. A IFNL3 levels significantly (unpaired t test t = 3.783, P = 0.0003) increased in high SLEDAI SLE patients (SLEDAI ≥ 4, N = 40; IFNL3 concentration 8.450 ± 1.263 pg/ml) than in low SLEDAI patients (SLEDAI = 0, N = 40; IFNL3 concentration 3.260 ± 0.5365 pg/ml). B IFNL3 levels not significantly different (unpaired t test t = 1.650, P = 0.103) between nephritis-positive patients (N = 40; IFNL3 concentration 4.645 ± 1.039 pg/ml) and nephritis-negative patients (N = 40; IFNL3 concentration 7.065 ± 1.036 pg/ml). (DOCX 194 kb) [file 13075_2018_1683_MOESM1_ESM.docx]

**Interferon-λ3/4 genetic variants and interferon-λ3 serum levels are biomarkers of lupus nephritis and disease activity in the Taiwanese**

Ji-Yih Chen^1^, Chin-Man Wang^2^, Tai-Di Chen^3^, Yeong-Jian Jan Wu^1^, Jing-Chi Lin^1^, Ling Ying Lu^4^ and Jianming Wu^5^

**Table S1**. Association of *IFN3/4* locus SNP haplotypes (rs8099917- ss469415590-rs12979860-rs4803217) with lupus nephritis among SLE patints

| Haplotype | Estimated Frequency (%) | | | Permutation | Logistic regression | | Logistic regression adjusted for sex and age | |
| --- | --- | --- | --- | --- | --- | --- | --- | --- |
|  | Nephritis^+^ | Nephritis^-^ | SLE cases | P value* | P value | OR (95% CI) | P value | OR (95% CI) |
|  | (N=561) | (N=452) | (N=1013) |  |  |  |  |  |
| T-TT-C-C | 92.59% | 88.81% | 90.90% | 0.0029 | 0.0029 | 1.61(1.18-2.19) | 0.0019 | 1.65(1.20-2.26) |
| G-ΔG-T-A | 4.45% | 7.73% | 5.92% | 0.0015 | 0.002 | 0.55(0.38-0.80) | 0.001 | 0.52(0.36-0.77) |
| Others | 3.83% | 4.78% | 4.25% |  | 0.3472 | 0.79 (0.49-1.28) | 0.3690 | 0.8 (0.49-1.3) |

**Figure S1.** Schematic illustration of *IFNL3/4* locus SNP locations. The sizes of exons and the distances between exons are indicated as base pairs (bps). The first exon (exon 1) of each gene starts from the ATG start codon and the last exon (exon 5) of each gene ends at the stop codon. The SNP rs8099917 is located in the *IFNL4* promoter region (3945 bps upstream of the translation starting site) and the SNP rs4803217 in the *IFNL3* 3’-UTR (52 bps downstream of the translation termination codon). Two other SNPs ss469415590 and rs12979860) are with the *IFNL4* gene.


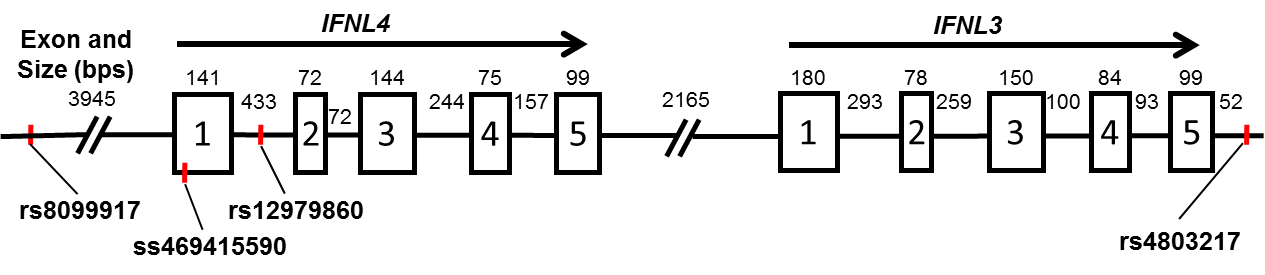


**Figure S2.** Pair-wise LD patterns of four *IFNL3/4* locus SNPs on Chromosome 19 show the coefficient of linkage disequilibrium D’ (red color) and the square of the correlation coefficient between two indicator variables γ^2^ (black color) of all subjects (A), SLE cases (B), and healthy controls (C), respectively. The darker colors indicate the stronger LD.

| (A)  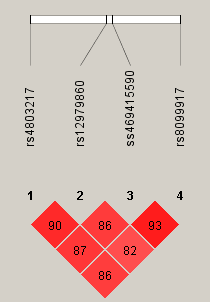 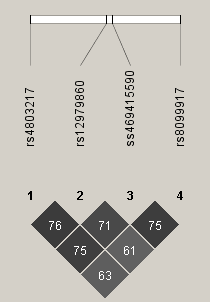 | (B)  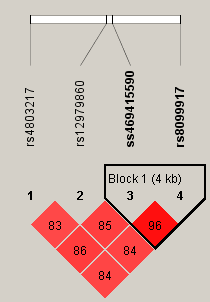 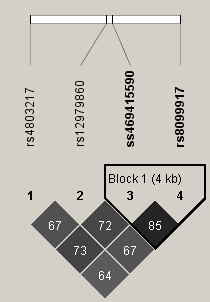 | (C)  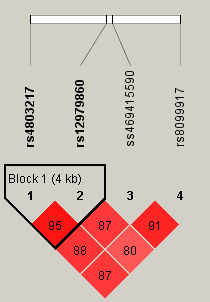 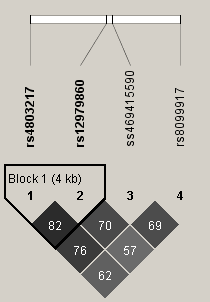 |
| --- | --- | --- |

**Figure S3.** Association of IFN3 levels with SLE disease activity (SLEDAI) in a replication cohort. **A).** IFNL3 levels were significantly (unpaired t-test t = 3.783, *P* = 0.0003) increased in high SLEDAI SLE patients (SLEDAI ≥ 4, N=40. IFNL3 concentrations: 8.450 ± 1.263 pg/ml) than in the low SLEDAI patients (SLEDAI = 0, N=40, IFNL3 concentrations: 3.260 ± 0.5365 pg/ml). **B).** IFNL3 levels were not significantly different (unpaired t-test t = 1.650, *P* = 0.103) between nephritis positive patients (N=40. IFNL3 concentrations 4.645 ± 1.039 pg/ml) and nephritis negative patients (N=40, IFNL3 concentrations: 7.065 ± 1.036 pg/ml).
